# Severe Chronic Mesenteric Ischemia in a Patient with Moyamoya Disease

**DOI:** 10.31662/jmaj.2024-0306

**Published:** 2025-03-21

**Authors:** Yohei Yamamoto, Kazuki Tsukuda, Ai Kazama, Yoshiki Wada, Hiroki Uchiyama, Toru Kikuchi, Toshifumi Kudo

**Affiliations:** 1 Division of Vascular Surgery, Department of Cardiovascular Surgery, Institute of Science Tokyo, Tokyo, Japan

**Keywords:** moyamoya disease, chronic mesenteric ischemia, celiac artery, superior mesenteric artery, celiacomesenteric trunk, revascularization

## Abstract

Moyamoya disease is a rare cerebrovascular occlusive disorder, and its natural course remains incompletely understood. Although rare, extracranial arterial lesions can develop in patients with moyamoya disease. We report the case of a 35-year-old Japanese woman with moyamoya disease who was referred to our department for the treatment of severe chronic mesenteric ischemia. She had a several-year history of postprandial abdominal pain and experienced two episodes of gastric ulcer perforation in the past year. Enhanced computed tomography revealed that the patient had a common trunk of the celiac and superior mesenteric arteries, which was occluded at its origin. The patient underwent an aorta to superior mesenteric artery bypass with a great saphenous vein graft. The postoperative period was uneventful, and the patient is now free of symptoms. The present case suggests that a patient with moyamoya disease can develop symptomatic mesenteric arterial lesions.

## Introduction

Moyamoya disease (MMD) is a rare cerebrovascular occlusive disorder, and its natural course remains incompletely understood. Although rare, extracranial arterial lesions can develop in patients with MMD.

Herein, we report the case of a patient with MMD who developed severe chronic mesenteric ischemia (CMI) due to occlusion of a common trunk of the celiac and superior mesenteric arteries.

## Case Report

**Figure 1.**
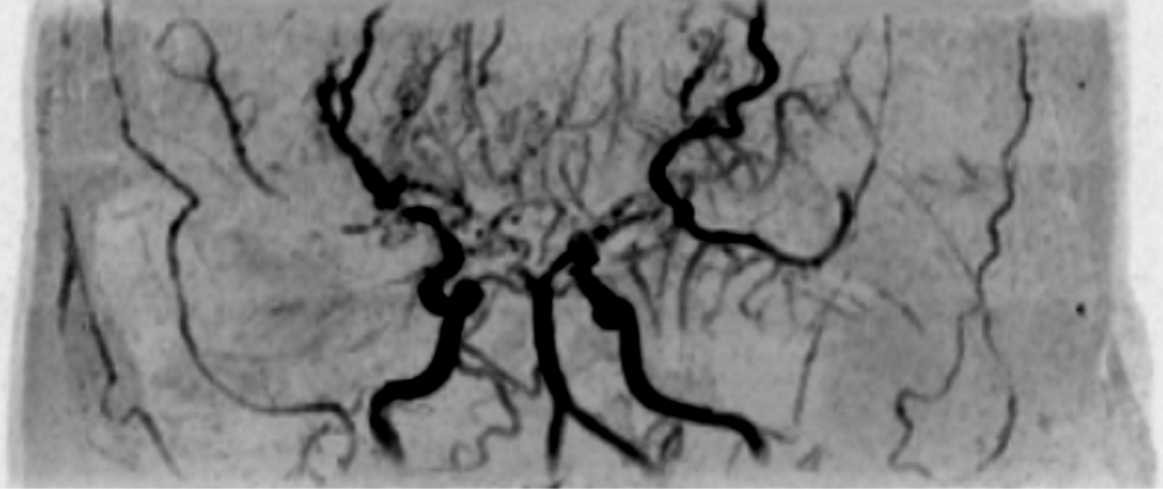
Magnetic resonance angiography of the intracranial arteries at the time of diagnosis.

**Figure 2.**
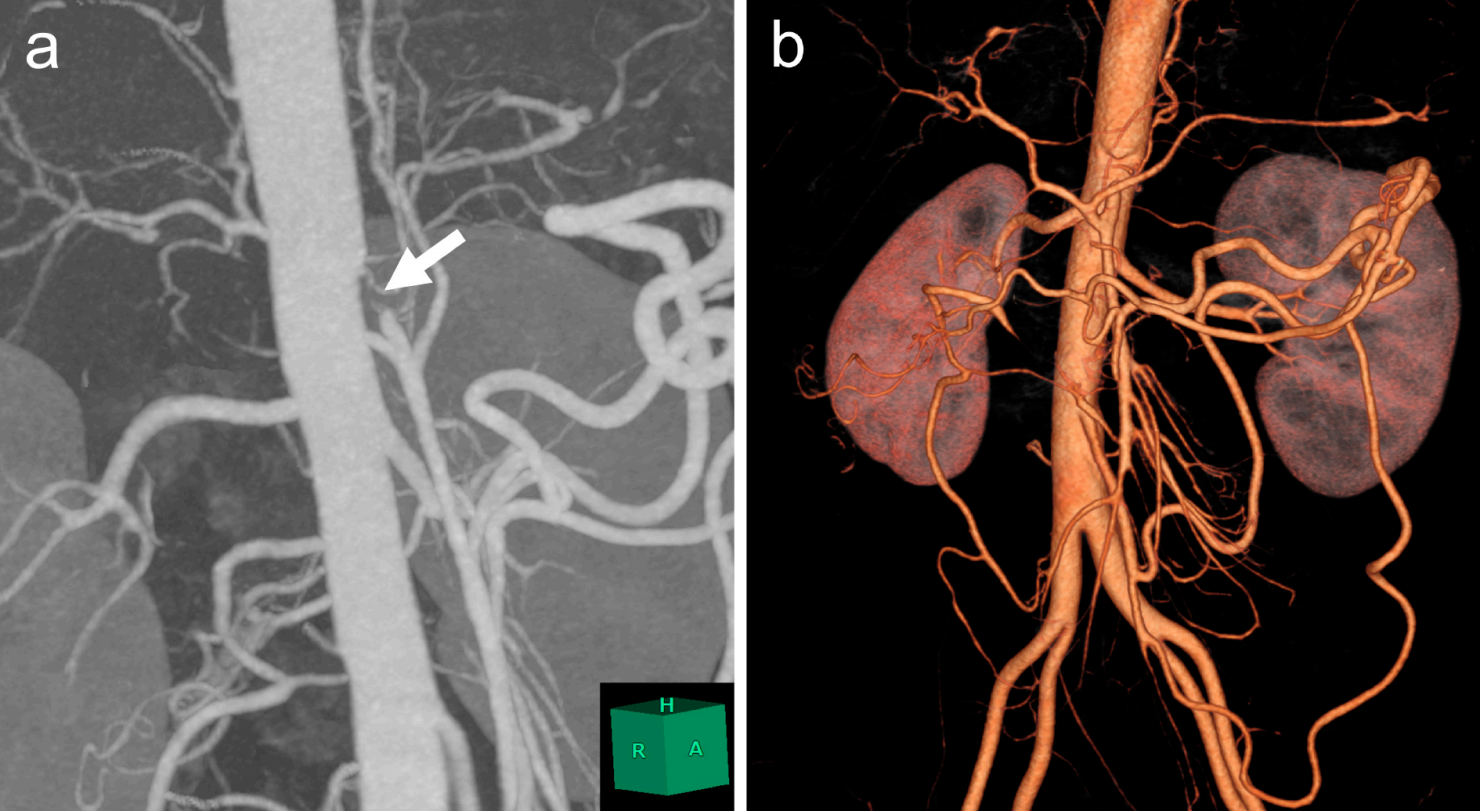
Computed tomography images showing occlusion of the common trunk of the celiac and superior mesenteric arteries (arrow) and the development of prominent collateral arteries via the inferior mesenteric artery. (a) Maximum intensity projection image. (b) Three-dimensional reconstruction image.

**Figure 3.**
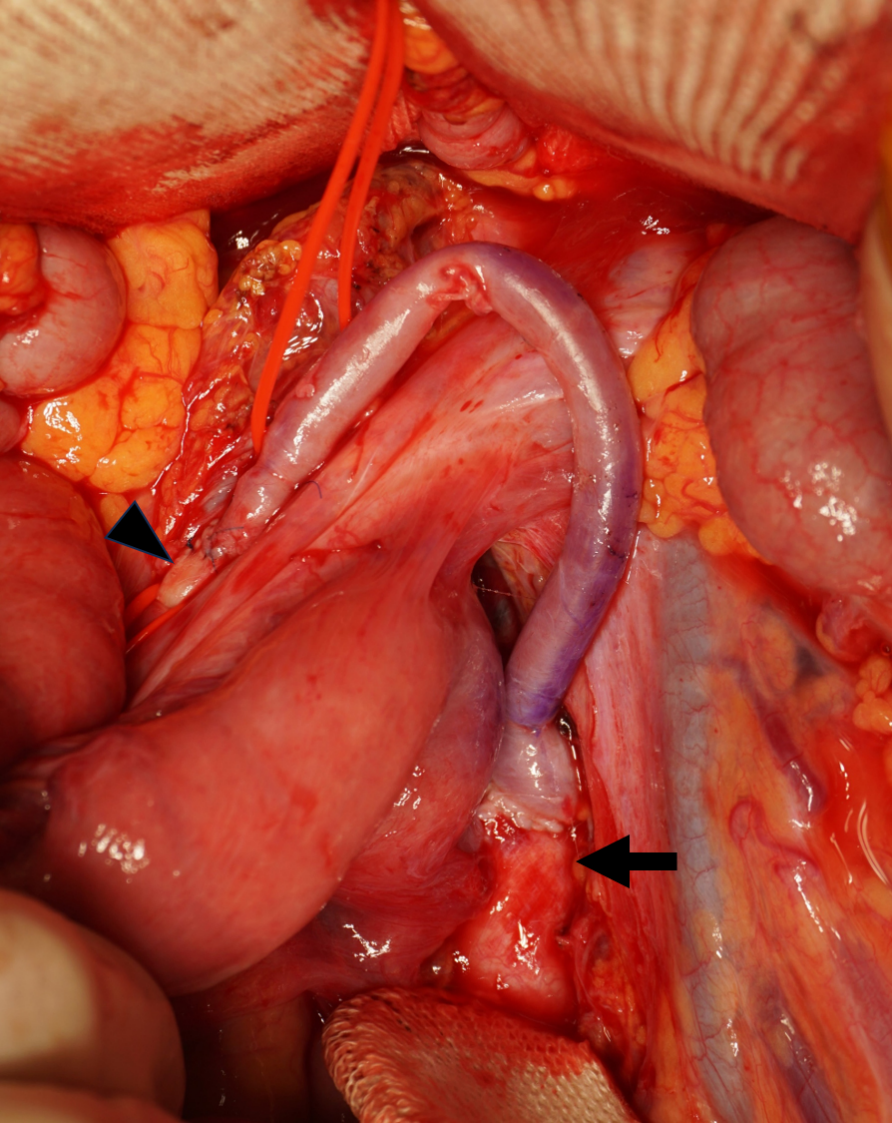
Intraoperative image showing the aorta (arrow) to superior mesenteric artery (arrowhead) bypass using a great saphenous vein graft.

**Figure 4.**
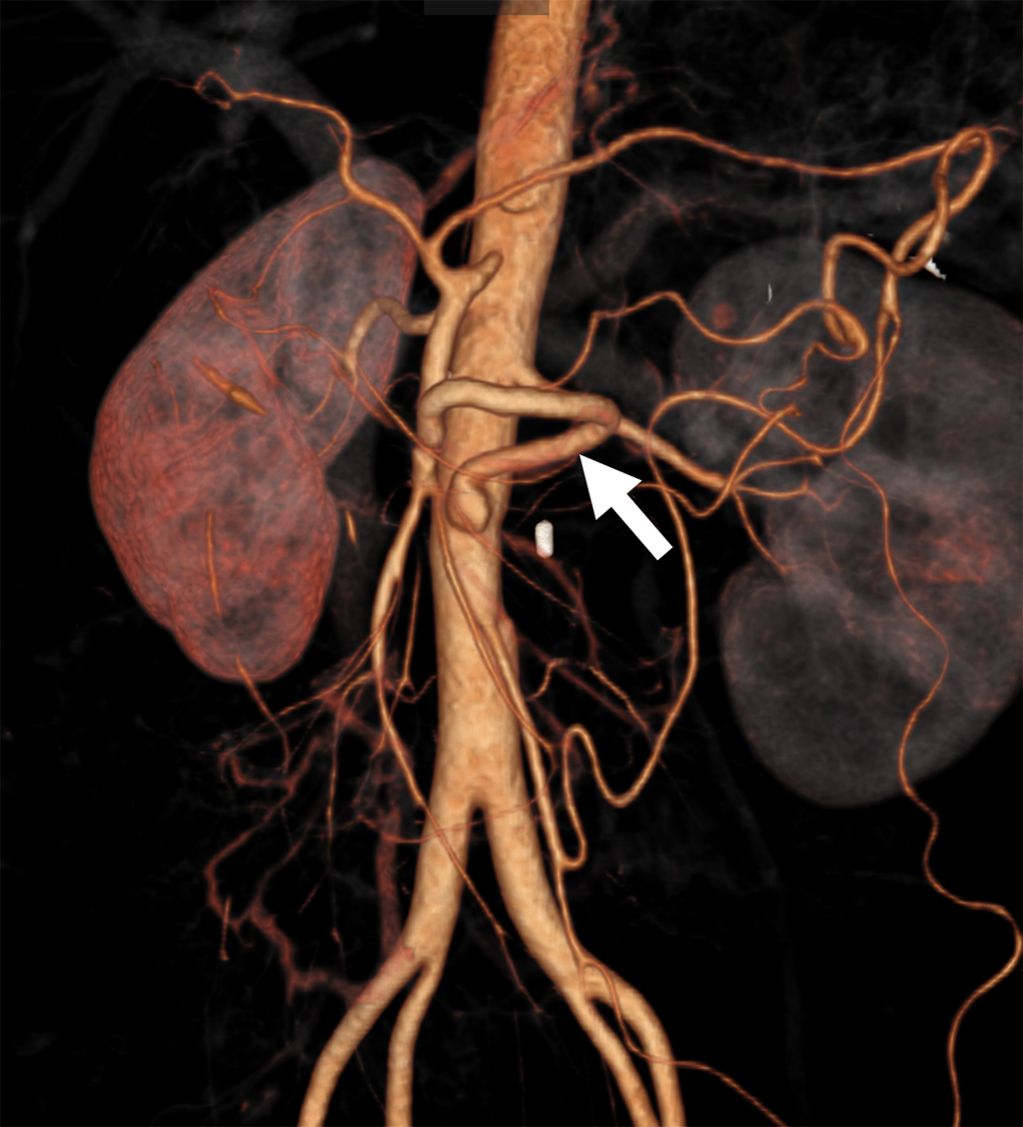
Postoperative computed tomography showing an aorta to superior mesenteric artery bypass (arrow).

A 35-year-old Japanese woman was referred to our department for the treatment of severe CMI. Her past medical history was significant for MMD which was diagnosed following investigations into episodic numbness in her upper extremities (Figure 1). Genetic testing was not available at the time of diagnosis. She underwent bilateral encephalo-duro-arterio-synangiosis procedures at the age of 21. Aside from MMD, she had no history of other underlying medical conditions. She had a several-year history of postprandial abdominal pain and experienced two episodes of gastric ulcer perforation in the past year, which were treated with omental patch repair. When she developed a gastric perforation, her body mass index was 18 kg/m^2^. The physical examination was otherwise unremarkable. Laboratory findings on admission showed anemia with a hemoglobin level of 9.0 g/dL. There was no evidence of systemic inflammation, metabolic disorders, or autoimmune diseases. Enhanced computed tomography revealed that the patient had a common trunk of the celiac and superior mesenteric arteries, which was occluded at its origin. No evidence of arterial compression by the median arcuate ligament was observed. It also demonstrated the development of prominent collateral arteries via the inferior mesenteric artery (Figure 2). No abnormalities were found in the other branches of the aorta. Because her gastrointestinal symptoms were strongly suspected to be associated with mesenteric ischemia, revascularization was indicated. Due to adhesions in the upper abdomen and the need for supra-celiac aortic clamp, in-situ revascularization or tissue sampling of the occluded artery was not considered. The patient underwent an aorta to superior mesenteric artery bypass with a great saphenous vein graft (Figure 3). The postoperative period was uneventful, and the patient was discharged seven days after surgery. Postoperative computed tomography demonstrated an intact bypass graft and reduced mesenteric collateralization (Figure 4). The patient has remained free of gastrointestinal symptoms since the operation.

## Discussion

MMD is a cerebrovascular disorder characterized by bilateral or unilateral stenosis or occlusion of the terminal portion of the internal carotid artery and the presence of an abnormal vascular network composed of collateral pathways at the base of the brain ^(1)^. Most pediatric patients present with recurrent ischemic events, whereas approximately half of adult patients experience intracranial hemorrhage ^(2)^. Our patient initially presented with cerebral ischemic symptoms and underwent indirect revascularization surgery more than 10 years before the onset of gastrointestinal symptoms. Several reports have described the extracranial vascular involvement of MMD. In 1991, an autopsy report demonstrated pathological changes in the extracranial arteries of patients with MMD, suggesting an underlying systemic arteriopathy associated with the disease ^(3)^. Supporting this, in 2011, the RNF213 gene was identified as a susceptibility gene for MMD ^(4), (5)^. The renal artery has been reported as the most commonly affected extracranial vessel, involved in 5%-8% of patients with MMD ^(6), (7)^. Some reports have described the association between MMD and early onset of coronary artery disease. Nam et al. ^(8)^ reported that 4.6% of patients with MMD, with a median age of 44 years, had symptomatic coronary artery disease. Involvement of other extracranial arteries, in contrast, is a very rare phenomenon in MMD.

In studies focusing on renal artery lesions, no stenosis was observed in the celiac or superior mesenteric arteries ^(6), (7)^.

On the other hand, Jee et al. ^(9)^ reported that four out of 63 patients with MMD exhibited significant stenosis in the celiac (n = 2) or superior mesenteric (n = 2) arteries. None of these patients were symptomatic.

Several vascular diseases can cause mesenteric arterial occlusive lesions, but our patient exhibited no clinical or laboratory manifestations of arteriosclerosis or vasculitis. Although histopathologic and genetic diagnoses are not confirmed, the clinical course of our patient strongly suggests that the mesenteric arterial lesion is an extracranial manifestation of MMD.

A common trunk of the celiac and superior mesenteric arteries is a rare vascular anatomical variation, which is known as the celiacomesenteric trunk. To our knowledge, there is no known association between this anatomical variation and MMD.

Occlusion of the celiacomesenteric trunk means the interruption of blood supply from both the celiac and superior mesenteric arteries. Therefore, our patient had severe ischemic symptoms.

In symptomatic patients with CMI, revascularization is indicated. Recently, less invasive endovascular treatment has been recommended as the first-line treatment. Open bypass surgery, however, offers durable patency and better symptomatic relief ^(10)^. Considering the extent of the disease and the patient’s life expectancy, we decided to perform open surgery, and a good result was achieved.

The present case suggests that a patient with MMD can develop symptomatic mesenteric arterial lesions. Open bypass surgery was effective in relieving the patient’s symptoms.

## Article Information

### Conflicts of Interest

None

### Author Contributions

All authors contributed to the conception and the acquisition of data for the work.

Yohei Yamamoto drafted the manuscript.

All authors have read and approved the final manuscript.

### Approval by Institutional Review Board (IRB)

IRB approval was not required for case reports.

### Informed Consent

Written informed consent was obtained from the patient for publication of this case report and accompanying images.

## References

[1] Kuroda S, Fujimura M, Takahashi J, et al. Diagnostic criteria for Moyamoya disease - 2021 revised version. Neurol Med Chir (Tokyo). 2022;62(7):307-12. 35613882 10.2176/jns-nmc.2022-0072 PMC9357455

[2] Kuroda S, Houkin K. Moyamoya disease: current concepts and future perspectives. Lancet Neurol. 2008;7(11):1056-66. 18940695 10.1016/S1474-4422(08)70240-0

[3] Ikeda E. Systemic vascular changes in spontaneous occlusion of the circle of Willis. Stroke. 1991;22(11):1358-62. 1750042 10.1161/01.str.22.11.1358

[4] Liu W, Morito D, Takashima S, et al. Identification of RNF213 as a susceptibility gene for Moyamoya disease and its possible role in vascular development. PLoS One. 2011;6(7):e22542. 21799892 10.1371/journal.pone.0022542 PMC3140517

[5] Kamada F, Aoki Y, Narisawa A, et al. A genome-wide association study identifies RNF213 as the first Moyamoya disease gene. J Hum Genet. 2011;56(1):34-40. 21048783 10.1038/jhg.2010.132

[6] Togao O, Mihara F, Yoshiura T, et al. Prevalence of stenoocclusive lesions in the renal and abdominal arteries in Moyamoya disease. AJR Am J Roentgenol. 2004;183(1):119-22. 15208124 10.2214/ajr.183.1.1830119

[7] Yamada I, Himeno Y, Matsushima Y, et al. Renal artery lesions in patients with Moyamoya disease: angiographic findings. Stroke. 2000;31(3):733-7. 10700512 10.1161/01.str.31.3.733

[8] Nam TM, Jo KI, Yeon JY, et al. Coronary heart disease in Moyamoya disease: are they concomitant or coincidence? J Korean Med Sci. 2015;30(4):470-4. 25829816 10.3346/jkms.2015.30.4.470 PMC4366969

[9] Jee TK, Yeon JY, Kim SM, et al. Prospective screening of extracranial systemic arteriopathy in young adults with Moyamoya disease. J Am Heart Assoc. 2020;9(19):e016670. 32954918 10.1161/JAHA.120.016670 PMC7792364

[10] Aboyans V, Ricco JB, Bartelink MEL, et al. Editor’s choice - 2017 ESC guidelines on the diagnosis and treatment of peripheral arterial diseases, in collaboration with the European Society for Vascular Surgery (ESVS). Eur J Vasc Endovasc Surg. 2018;55(3):305-68. 28851596 10.1016/j.ejvs.2017.07.018

